# Clinical role, optimal timing and frequency of serum infliximab and anti-infliximab antibody level measurements in patients with inflammatory bowel disease

**DOI:** 10.1371/journal.pone.0172916

**Published:** 2017-03-31

**Authors:** Renáta Bor, Klaudia Farkas, Anna Fábián, Anita Bálint, Ágnes Milassin, Mariann Rutka, Mária Matuz, Ferenc Nagy, Zoltán Szepes, Tamás Molnár

**Affiliations:** 1 First Department of Medicine, University of Szeged, Szeged, Hungary; 2 Department of Clinical Pharmacy, Faculty of Pharmacy, University of Szeged, Szeged, Hungary; Katholieke Universiteit Leuven Rega Institute for Medical Research, BELGIUM

## Abstract

**Background:**

Serum infliximab (IFX) and antibody-to-infliximab (ATI) levels are objective parameters, that may have a great role in the therapeutic decisions during maintenance biological therapy.

**Research design and methods:**

48 inflammatory bowel disease patients receiving maintenance IFX therapy were prospectively enrolled and divided into adequate (complete remission N = 20) and inadequate responder (partial response, loss of response, dose escalation; N = 28) groups. Blood samples were collected just before (trough level, TL) and two (W2aTL) and six weeks (W6aTL) after the administration of IFX.

**Results:**

Single measurement of ATI titer was insufficient for predicting therapeutic response due to transient expression of ATI, however, using the three points’ measurements, significant difference has been detected between the adequate and inadequate responder group (5.0% vs 35.7%; p = 0.016). The mean value of TL was significantly higher in the adequate responder group (3.11±1.64 vs.1.19±1.11; p<0.001) without further difference on the second and sixth week. Sensitivity and specificity for predicting the therapeutic response were 85.0% and 71.4% based on the cut-off value of TL 2.0 μg/ml.

**Conclusion:**

Simultaneous measurement of serum IFX level prior to administration of regular IFX infusion and ATI titers significantly increase the diagnostic accuracy for the therapeutic decision in patients uncertainly responding to the therapy. The measurement of W2aTL and W6aTL levels did not result in further improvement in the prediction of therapeutic response.

## Introduction

The introduction of biological treatment has made a major break through in the management of inflammatory bowel disease (IBD). However, a substantial number of patients show only partial response, and approximately 20–45% of the primary responders show loss of efficacy [1–4]. Cessation of therapy or switching to another biological drug currently depends mainly on subjective clinical judgement. Serum infliximab (IFX) and antibody-to-infliximab (ATI) levels are objective parameters that may help in the therapeutic decisions during maintenance biological therapy. Results of recent studies suggest that serum IFX concentration predicts long-term clinical response [5]. In ulcerative colitis (UC), detectable IFX trough level (TL) is associated with higher rate of clinical remission and endoscopic improvement and with lower risk of colectomy [6]. ATI is reported to develop up to 60% of IBD patients during maintenance IFX therapy [7,8]. The presence of ATI is associated with lower serum IFX levels, higher rate of infusion reactions and loss of response, and it may shorten the effect of IFX infusions [7,9].

Despite the proven importance of serum IFX and ATI levels in the prediction of clinical response, it is still not clearly defined when and how frequently we have to measure these titers. Therefore, the aim of this study was to determine the optimal timing and frequency of serum IFX and ATI measurements. We aimed to assess the correlation between serum IFX and ATI levels and response to IFX therapy and to determine the accuracy of serum drug concentration measurement in the prediction of the long-term clinical response.

## Patients and methods

Forty-eight consecutive, adult IBD patients receiving IFX maintenance therapy were prospectively enrolled between March 2014 and October 2015 in a Hungarian tertiary referral medical center. All patients received detailed written and verbal information about the investigation, and they consented to participation in this study. The protocol was approved by the Regional and Institutional Human Medical Biological Research Ethics Committee of the University of Szeged (SZTE: 169/2011). The study was carried out under the declaration of Helsinki.

IFX was administered intravenously with maintenance dosage of 5 or 10 mg/kg every 8 weeks as monotherapy or in combination with azathioprine, 5-aminosalicylates and/or corticosteroids. In our study no distinction has been made between original and biosimilar IFX, because previous studies did not find any difference in terms of efficacy, safety and immunogenicity between the original and biosimilar agent [10–12]. Patients were divided into adequate and inadequate responder groups based on their clinical response at inclusion, which was determined with partial Mayo Score (pMayo) and Crohn's Disease Activity Index (CDAI). Adequate response was defined as complete clinical remission with pMayo score ≤2 or CDAI score <150 during the previous 6 months on maintenance therapy. Patients were categorized into the inadequate responder group, if: 1) they partially responded to 5 mg/kg dose IFX therapy (a decrease in pMayo score of ≥3 points or in CDAI score of 100 point from baseline); 2) dose escalation was required (10 mg/kg body weight) during the previous 6 months; 3) loss of response occurred at inclusion. The baseline was the time when patient received the first IFX infusion, so response correspond to changes of scores during the biological therapy.

Blood samples were collected for serum IFX and ATI measurements at inclusion–immediately prior the administration of regular maintenance IFX infusion (trough level, TL)–, as well as 2 (W2aTL) and 6 weeks (W6aTL) afterwards. Serum samples were tested by quantitative enzyme-linked immunosorbent assay (ELISA) with LISA-Tracker (Theradiag, France). The detectable serum IFX level was 0.1 μg/ml. In case of LISA TRACKER, the measurement range was 10 to 200 ng/mL for antibodies and > 10 ng/mL was considered to be positive. At the end of the 6-months follow-up the response to IFX therapy was re-evaluated by using pMayo and CDAI scoring system.

Patients' demographic data, clinical characteristics, previous surgery and concomitant medications were collected using MedSolution medical recorder. Statistical tests were performed using R statistical software version 3.3.1 (R Foundation) and SPSS software version 24 (SPSS Inc., Chicago, Illinois, USA), values of p<0.05 were considered statistically significant. Differences in continuous variables such as serum IFX level, disease duration, age at the diagnosis were assessed with Welch Two Sample t-test. Fisher's Exact Test were used to compare the proportion of categorical variables in the adequate and inadequate responder group (ATI positivity, type of disease, gender, concomitant treatment). The cut-off levels were determined by receiver operating characteristic (ROC) curve analysis, which used clinical remission as a classification variable to calculate the sensitivity, specificity and area under the ROC curve (AUC). Multivariate models were constructed using logistic regression (Overall model fit was described with Nagelkerke R^2^, and the goodness-of-fit by use of Hosmer and Lemeshow Test). Descriptive statistics were reported as mean, median and interquartile range, categorical variables were expressed as percentage.

## Results

### Patient characteristics at inclusion

The adequate responder group consisted of 20 patients being in sustained clinical remission on maintenance IFX therapy. The inadequate responder group (N = 28) was heterogeneous: 8 patients showed chronic activity during the last 6 months of IFX maintenance therapy with mild (N = 7) or moderate (N = 1) disease activity. Fourteen patients required dose escalation (10 mg/kg) in the last 6 months: 3 of them were in remission, in 10 cases mild and in one case moderate activity was observed at the time of inclusion. Six patients had relapse at the time of the first sampling (TL).

Forty-two patients received original and 6 patients received biosimilar IFX (4 inadequate and 2 adequate responder). IFX monotherapy was applied in only one third of patients (N = 16), in the remaining cases it was complemented by azathioprine (N = 26), 5-aminosalicylates (N = 10), local (N = 3) and/or systemic corticosteroids (N = 6). There was no significant proportional variance regarding gender, mean age at the diagnosis and disease duration between the groups (Table 1). Rate of Crohn’s disease (CD) patients were higher in the inadequate responder group, but the difference was not statistically relevant, and the demographic and clinical characteristics between UC and CD patients did not differ significantly.

**Table 1:** Clinical and demographic data of 48 enrolled inflammatory bowel disease patients.

**Clinical and demographic data of patients (N = 48)**		
	Adequate responder (N = 20)	Inadequate responder (N = 28)
**Female/male (N°)**	10/10	13/15
**UC/CD (N°)**	9/11	7/21
**Ulcerative colitis**		
• **pancolitis**	4 (20%)	4 (14.3%)
• **left-sided colitis**	5 (25%)	3 (10.7%)
**Crohn’s Disease**		
• **ileal (L1)**	-	2 (7.1%)
• **colonic (L2)**	4 (20%)	11 (39.3%)
• **ileocolonic (L3)**	7 (35%)	8 (28.6%)
• **non stricturing, non penetrating (B1)**	4 (20%)	8 (28.6%)
• **stricturing (B2)**	2 (10%)	3 (10.7%)
• **penetrating (B3)**	5 (25%)	10 (35.7%)
• **perianal (p)**	7 (35%)	9 (32.1%)
**Age at the diagnosis (years)**	25.00±9.21	26.29±9.78
**Disease duration (years)**	9.14±5.32	7.40±5.35
**Duration of infliximab therapy**		
• **< 1 year**	7 (35%)	5 (17.9%)
• **1–2 years**	8 (40%)	11 (39.3%)
• **> 2 years**	5 (25%)	12 (42.9%)
**Previous surgery (N°; %)**	7 (35%)	14 (50%)
• **Seton drainage**	6 (30%)	9 (32.1%)
• **Intraabdominal fistula**	-	2 (7.1%)
• **Ileocecal resection**	1 (5%)	3 (15%)
• **Right hemicolectomy**	-	1 (3.6%)
**Concomitant therapy (N°; %)**		
• **Azathioprine**	14 (70%)	12 (42.7%)
• **Local steroid**	1 (5%)	2 (7.1%)
• **Systemic steroid**	1 (5%)	5 (17.9%)
• **5-aminosalycilate**	7 (35%)	3 (15%)

UC-ulcerative colitis; CD–Crohn’s Disease

### Serum IFX levels

**Fig 1:**
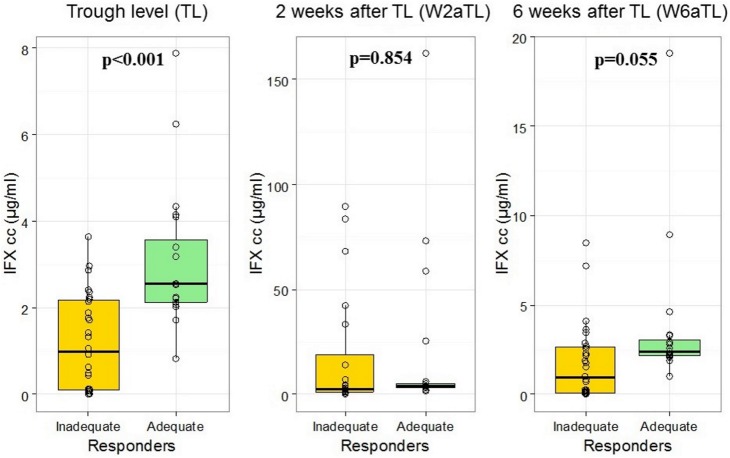
Serum IFX levels immediately prior the administration of regular maintenance infliximab (IFX) infusion (trough level, TL), as well as 2 (W2aTL) and 6 weeks (W6aTL) afterwards in the adequate and inadequate responder group.

**Fig 2:**
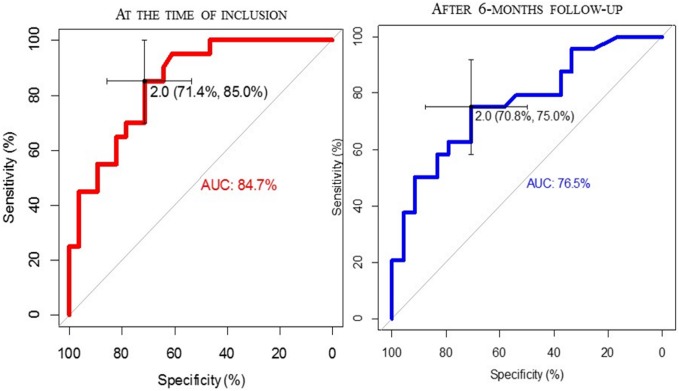
ROC analysis of IFX trough levels (TL) associated with current and long-term response.

**Fig 3:**
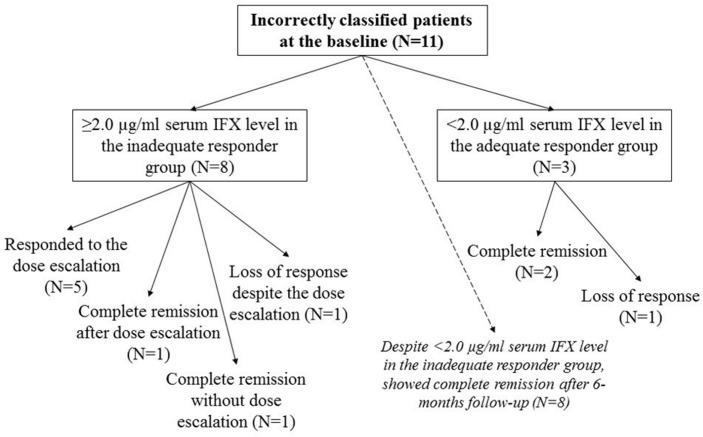
Incorrectly classified cases by serum infliximab trough levels after 6-months follow-up.

Serum IFX level was measured three times (TL, W2aTL, W6aTL) during the administration of regular maintenance infusion. The mean value of serum TL was significantly higher in the adequate vs. inadequate responder group (3.11±1.64 vs.1.19±1.11; p<0.001). Mean IFX levels did not differ between the groups at week 2 (18.87±39.05 vs. 16.99±27.65; p = 0.854) and week 6 (3.69±3.96 vs. 1.74±2.15; p = 0.055) (Fig 1). Therefore, W2aTL and W6aTL levels were not suitable for the prediction of therapeutic response. According to ROC analysis, the cut-off value of TL for predicting therapeutic response was 2.0 μg/ml with 85.0% sensitivity and 74.1% specificity. The AUC was 84.7% (Fig 2). In the inadequate responder group, ≥2.0 μg/ml TL was measured in 8 cases: six patients received intensified IFX therapy (10mg/kg every 8 weeks) from which five patients responded to the dose escalation. One of the three adequate responders with low IFX level and ATI positivity developed allergic reaction, the remaining two patients with low IFX level without ATI positivity were in clinical remission. (Fig 3) The results of multivariate analysis (TL, W2aTL, W6aTL levels and ATI positivity) performed by logistic regression revealed prediction rate of 85.4% for the current response (Table 2). It showed high similarity with the results of ROC analysis, which assessed only the TL. Therefore, measurement of W2aTL and W6aTL levels did not improve the accuracy of prediction of therapeutic response.

**Table 2:** Results of logistic regression analysis for prediction current (at inclusion) and long-term response (after 6-months follow-up).

**LOGISTIC REGRESSION FOR PREDICTION CURRENTS RESPONSE** **(Overall model fit: Nagelkerke R^2^ = 0.668; Goodness-of-fit: Hosmer and Lemeshow Test p = 0.300;** **Classification table: Correctly predictions = 85.4%)**						
	**B**	**S.E.**	**df**	**p**	**OR = Exp(B)**	**95% CI for OR**
**TL**	1.81	0.64	1	0.005	6.137	1.75–21.53
**W2aTL**	-0.07	0.03	1	0.013	0.928	0.88–0.99
**W6aTL**	0.41	0.33	1	0.217	1.506	0.79–2.88
**ATI**	0.18	1.44	1	0.900	1.198	0.07–20.03
**Constant**	-3.94	1.37	1	0.004	0.019	
**LOGISTIC REGRESSION FOR PREDICTION LONG-TERM RESPONSE** **(Overall model fit: Nagelkerke R^2^ = 0.438; Goodness-of-fit: Hosmer and Lemeshow Test p = 0.221;** **Classification table: Correctly predictions = 77.1%)**						
	**B**	**S.E.**	**df**	**p**	**OR = Exp(B)**	**95% CI for OR**
**TL**	1.26	0.48	1	0.008	3.515	1.38–8.96
**W2aTL**	-0.05	0.02	1	0.020	0.953	0.92–0.99
**W6aTL**	0.15	0.22	1	0.505	1.160	0.75–1.8
**ATI**	0.46	0.99	1	0.644	1.585	0.23–11.18
**Constant**	-1.99	0.87	1	0.022	0.136	

B: regression coefficient; S.E.: standard error; df: degree of freedom; OR: odds ratio; CI: confidence interval; TL: serum infliximab [IFX] trough level; W2aTL: serum IFX level 2 weeks after TL; W6aTL: serum IFX level 6 weeks after TL; ATI: antibody-to-infliximab

Response to biological therapy was reevaluated after the 6-months follow-up. Five inadequate responders were re-classified into the adequate responder group. In one of them optimal serum IFX level was measured without ATI positivity. The clinical data of the patient suggested an ongoing infection at the time of the inclusion, which resolved after the administration of antibiotics. In two cases with dose escalation at inclusion, serum W2aTL level was higher than 2 μg/ml, but the drug concentration dropped rapidly to an almost undetectable level by week 6. In these cases, ATI expression was also detectable, which suggests an accelerated drug elimination from the circulation. Despite TL not reaching the cut-off value (1.71 μg/ml and 0.83 μg/ml), two patients showed complete clinical remission. No ATI expression was detectable in these cases. (Fig 3) ROC analysis was performed to calculate the accuracy of previously determined 2.0 μg/ml cut-off value of TL for prediction of long-term therapeutic response. Serum IFX levels showed better correlation with the current status than with the long-term efficacy. The sensitivity and specificity in the prediction of long-term response was 70.8% and 75.0% (AUC: 76.5%). (Fig 2) Prediction rate in logistic regression model was 77.1%, which correlated with the results of ROC analysis. (Table 2)

### ATI positivity

**Fig 4:**
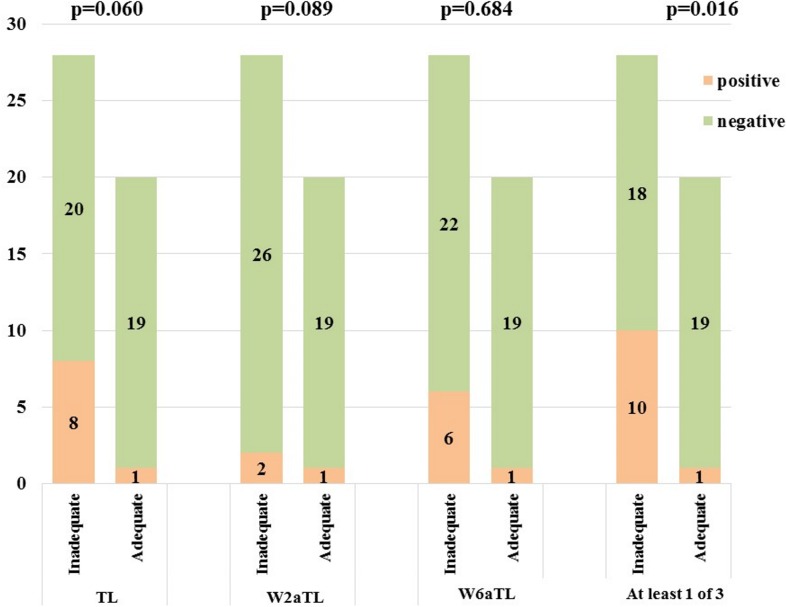
Proportion of ATI positivity in the adequate and inadequate responder groups.

**Fig 5:**
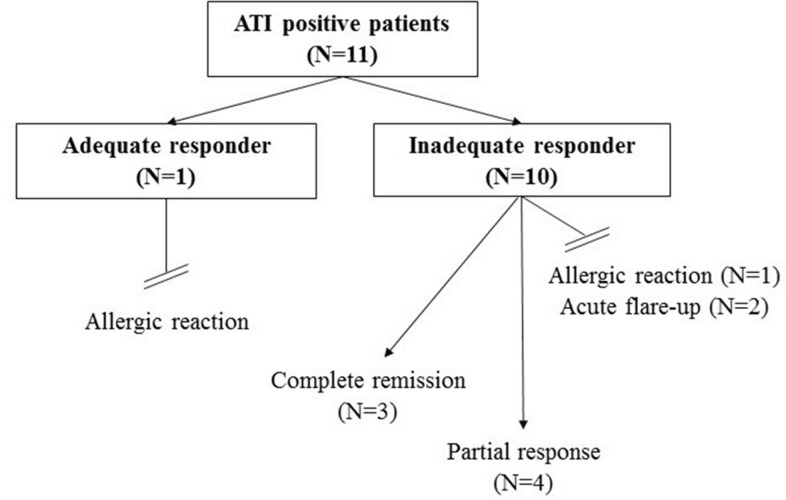
Antibody-to-infliximab (ATI) positive cases after 6-months follow-up.

ATI was identified in 11 patients with low serum IFX levels (<1 μg/ml). In 9 cases antibodies were not detectable in all of the three consecutive blood samples, suggesting that the expression of ATI in the blood was transient. Single sampling of ATI showed a nonsignificant trend for the correlation with the therapeutic response. The proportion of ATI positivity in the adequate and inadequate responder groups was 5.0% vs. 28.5% (p = 0.060) immediately prior administration of regular maintenance IFX infusion, but two and six weeks after the biological therapy it was 5.0% vs. 7.1% (p = 0.684) and 5.0% vs. 21.0% (p = 0.089). Using the three points’ measurements, ATI expression showed significant difference between the adequate and inadequate responder groups (5.0% vs 35.7%; p = 0.016). (Fig 4) In one of the ATI positive, adequate responder patients, allergic reaction occurred during the subsequent regular IFX infusion. After the 6-months follow-up clinical remission was achieved in three cases, when IFX 5 mg/kg therapy was combined with perianal surgical treatment (seton drainage). Four patients showed partial response to biological therapy. In three cases acute flare-up was observed, requiring surgery or switching to another biological agent. (Fig 5)

## Discussion

Our prospective study of IBD patients receiving maintenance IFX therapy aimed to determine the optimal timing and frequency of serum IFX and ATI measurements for the prediction of therapeutic response. We found that determination of serum IFX level prior to the administration of regular IFX infusion and ATI positivity showed strong correlation with disease activity and predicted the at least 6-months-long response. Measurement of serum IFX 2 or 6 weeks after the infusion did not result in further elevation in the prediction rate.

Most studies have suggested that the measurement of serum IFX levels immediately after induction or during maintenance therapy may help to optimize biological treatment since it may help to decide about the necessity of dose escalation, cessation of therapy or the switching to another biological drug. A multicenter retrospective study of 16 severe and 16 moderately severe UC patients has detected significantly lower IFX TL in the acute severe UC group compared to the moderately severe group [13]. The post hoc analysis of ACCENT I study carried out by *Cornillie et al*. revealed higher median week 14 serum IFX TL in patients with sustained response to scheduled maintenance IFX 5 mg/kg without dose escalation compared to those who lost response during the 54-week follow-up: 4.0 vs 1.9 μg/ml. The optimal cut-off value for predicting therapeutic response was ≥3.5 μg/ml at week 14 [14]. This study did not confirm whether serum IFX level predicts therapeutic response in patients receiving IFX 10 mg/kg. On the contrary, *Paul et al*. found significant increase in IFX TL (considered as a positive delta IFX) in patients who responded to dose escalation. The delta IFX after drug optimization was 2.2 μg/ml versus 0.2 μg/ml in the responder and nonresponder groups. The 0.5 μg/ml cut-off delta IFX was independently associated with mucosal healing (likelihood ratio 2.02; 95% CI, 1.01–4.08; p = 0.048) [15]. In the majority of previous studies timing of measurement is not uniform. Week 14, after induction therapy is one of the most accepted sampling time, but it is applicable only in case of newly administered IFX therapy to predict long-term response especially in questionable cases. In other studies, samples were taken in various times (at week 22, 30, 52 or 54) or at the time of relapse. Currently there is no evidence-based recommendation about the optimal timing of measurement of serum IFX levels in patients who receive maintenance biological therapy. The meta-analysis of 22 studies carried out by *Moore et al*. in 2016 has found that the >2μg/ml cut-off trough IFX level during maintenance therapy is associated with greater probability of clinical remission (risk ratio RR 2.9, 95% CI 1.8–4.7, p<0.001) and mucosal healing (RR 3.0, 95% CI 1.4–6.5, p = 0.004) [16]. The main limitation of this analysis was that inclusion criteria and the time of sampling was not uniform. In our study we found that the measurement of serum IFX level was effective in the prediction of therapeutic response only prior to the administration of regular IFX infusion, and that multiple sampling (W2aTL and W6aTL) did not result in further increase in the prediction rate. The 2.0 μg/ml cut-off IFXw0 value showed slightly better correlation with the current condition than with long-term response: sensitivity and specificity were 71.4% and 85% vs. 70.8% vs. 75.0%. It is important to highlight that partial response or loss of response were observed only in three patients with ≥2.0 μg/ml TL during 5 or 10 mg/kg IFX maintenance therapy. It suggests that the measurement of serum IFX levels may be a great predictor of response both in case of normal dose of IFX therapy and after dose escalation.

Antibody formation against IFX may be observed in 60% of patients with episodic administration and in 6–25% of cases with scheduled biological therapy [8,17]. Based on the results of *Ungar et al*. ATI-free survival can be achieved by 42% of patients by 4-years follow-up, and in 90% of the cases the antibody appears within the first 12 months of therapy [18]. Use of concomitant immunosuppressants such as azathioprine and methotrexate may result in a 50% reduction in the risk of developing ATI (p<0.00001) [19]. The assessment of 13 studies with data of 1378 patients found that the risk of loss of response to IFX therapy in ATI positive IBD patients is elevated: risk ratio was 3.2 (95% CI: 2.0–4.9, p<0.0001), when compared with the ATI negative group [9]. ATI formation was associated with lower serum IFX levels. The standardized mean difference in trough serum IFX levels between groups was −0.8 (95% CI: −1.2, −0.4, p<0.0001). Furthermore, the presence of ATI increases the rate of infusion reactions and serum sickness–like reactions [20]. In the study of *O’Meara et al*. the risk ratio of any acute infusion reaction and severe infusion reactions was 2.4 (95% CI: 1.5–3.8, p<0.001) and 5.8 (95% CI: 1.7–19, p = 0.004) in ATI positive patients when compared with patients without ATI, but the rate of delayed hypersensitivity reactions did not differ significantly between the groups [7]. *Baert et al*. determined that the optimal cut-off serum ATI concentration for the prediction of shorter duration of response and infusion reactions is 8.0 μg/ml [21]. In our study ATI formation was observed in 11 patients, and was associated with lower serum IFX levels in all of the cases. The proportion of ATI was higher in the inadequate responder group, but only the three points' measurement was able to establish significant difference between the groups. ATI formation may increase the risk of loss of response, but could not exclude the opportunity of clinical remission particularly after dose escalation or during combined surgical and medical therapy. Therefore, in case of ATI positivity overall assessment of symptoms, serum IFX levels and therapeutic response considering subjective judgment is required.

Our results suggest that the simultaneous measurement of IFX TL and ATI titers significantly increase the diagnostic accuracy for the therapeutic decision in uncertainly responding patients. The measurement of W2aTL and W6aTL levels does not improve further the accuracy of the prediction of therapeutic response, but results in substantially elevated costs. The expression of ATI in the circulation may be transient, therefore single sampling is supposed to be insufficient for predicting the therapeutic response. It increases the risk of loss of response, but does not exclude the optimal response to normal or escalated dose of IFX. We recommend simultaneous assessment of serum IFX and ATI levels together with the clinical condition of patients. Clinical response based on the subjective judgment of the attending physician always takes priority over the results of measurement.

## Supporting information

S1 File (XLSX)
